# Examining inequality in utilisation of health management services for the elderly in rural Henan China

**DOI:** 10.1186/s12913-020-05630-7

**Published:** 2020-08-17

**Authors:** Huan Wu, Yaqing Liu

**Affiliations:** 1 School of Medicine and Health Management, Tongji Medical College, Huazhong University of Science and Technology, Wuhan, Hubei China; 2 Management Institute, Xinxiang Medical University, Xinxiang, Henan China

**Keywords:** Health management services, The elderly, Inequality, Concentration index, Decomposition, China

## Abstract

**Background:**

The health management plays an important role in improving the quality of life of the elderly and relieving the pressure of health resource consumption. This study aims to assess the income-related inequality in utilisation of health management services (HMS) for the elderly and the contribution of the related factors to inequality in rural Henan China.

**Methods:**

The data from 2015 Henan Rural Residents Health Survey with 1403 elderly people as the final sample were used for analysis. The concentration index (CI) was used to measure inequality in HMS utilisation for the elderly (no HMS, health assessment, physical examination, auxiliary examination, and health guidance). The decomposition of CI was adopted to explain the contribution of various determinants to inequality in HMS utilisation for the elderly.

**Results:**

No HMS utilisation was disproportionately concentrated among the poor (*CI* = − 0.0730*, p =* 0.0155), utilisation of physical and auxiliary examination was disproportionately concentrated among the rich (*CI* = 0.0575*, p* = 0.0448; *CI* = 0.0811*, p* = 0.0044). In addition, the pro-poor effects of health assessment and guidance utilisation were not statistically significant (*CI* = − 0.0173, *p* = 0.4617; *CI* = − 0.0213, *p* = 0.3900). The results of CI decomposition revealed that household income and family size made positive contributions to inequality while social medical insurance, gender, marital status, and age made negative contributions to inequality. The improved service satisfaction with village clinics could reduce inequality in HMS utilisation, while the improved service satisfaction with township hospitals could increase inequality in HMS utilisation.

**Conclusions:**

Although HMS for the elderly is provided free of charge, its accessibility remains pro-rich due to various factors. Policy makers should adopt effective interventions to resolve the contradiction between these factors and the utilisation of HMS, and redress inequality in the utilisation of HMS.

## Background

Population aging is closely related to the increased risk of diseases, such as heart disease, stroke, and chronic respiratory disorders. These diseases not only reduce the quality of life of the elderly but also consume a large amount of health resources. Therefore, due to population aging, various countries pay more and more attention to the health of the elderly, and many countries have established health management plans for seniors [1–7].

As a populous country, China faces the challenge of aging, which occurs at rates faster than those in many countries [8]. About 166.58 million people (11.9%) were 65 years or over in 2018, and this number was 53.51 million (3.4%) more than 10 years ago [9]. The development of industrialisation has led to an increasing number of young agricultural population migrating to cities which makes the aging problem in rural areas more serious in China [10]. To detect health risks early, prevent and control diseases, reduce medical expenses, and develop and maintain the healthy life for the elderly, the Central Committee of the Communist Party of China and the State Council explicitly added health management for the elderly into the national basic public health services in the context of the reform of China’s medical care system (2009). Health management services (HMS) for the elderly include four components [11]: (I) health assessment conducted by evaluating the basic health level, self-care ability, lifestyle, past disease history, and common symptoms and treatment of chronic diseases through enquiring the elderly; (II) physical examinations, including measurement of body temperature, pulse, blood pressure, height, and weight; routine examination of skin, superficial lymph nodes, the heart, lungs, and abdomen; and rough judgment of vision, hearing, and exercise; (III) auxiliary examinations, including blood routine, urine routine, fasting blood glucose, blood lipid, liver function (serum glutamic oxaloacetic transaminase, glutamic pyruvate transaminase and total bilirubin), renal function (serum creatinine and blood urea nitrogen), and electrocardiogram detection; and (IV) health guidance provided by health workers to the elderly based on the results of health evaluation. The Chinese government requires primary health care facilities to provide free HMS once a year for people aged 65 years and over.

In general, health management improves the physical health of the elderly [12, 13]. However, challenges still exist; unequal access to HMS for the elderly is one of the most important issues to be addressed. Some studies show that the utilisation of HMS for the elderly is associated with gender, income, education level, occupation and so on [14, 15]. However, few studies have examined inequality in HMS utilisation for the elderly; seniors in rural areas are more likely to be ignored. Although some scholars have reported that there is inequality for the elderly to utilise HMS in other countries, we cannot be sure that the findings are consistent with those in China [16, 17].

To fill previous research gaps, this study used concentration index (CI) to assess income-related inequality in HMS utilisation among the elderly in rural Henan, China. It also used CI decomposition to evaluate the effects of various factors on inequality in HMS utilisation among the elderly. Results could help policymakers to identify disadvantaged groups in HMS utilisation, find out the influencing factors of inequality in HMS utilisation, and provide basis for further improvement of health management policies for the elderly.

## Methods

### Data sources

Data from 2015 Henan Rural Residents Health Survey (HRRHS 2015) were analyzed. The survey used a multistage sampling design to obtain samples. Thirty-five counties were randomly selected according to the level of their economic development. Four townships were randomly selected from each county. At least 20 households were randomly selected from each township. HRRHS 2015 is a survey on health security system, flow direction of patients, and utilisation of basic public health services in rural areas of Henan. All information was presented in a structured questionnaire and collected through face-to-face interviews. This survey was approved by the Ethics Committee of Xinxiang Medical University (The reference number is XYLL—2,015,005). Before the formal survey, all interviewers were trained by a specialist and passed the examination. The purpose of the survey was explained to all respondents, and those who agreed to participate were asked to sign an informed consent form.

After excluding invalid data, 2983 households and 8487 individuals were included in HRRHS 2015. This study focused on the elderly (≥65 years) who have lived locally for more than 6 months. The final sample consisted of 1403 elderly people in HRRHS 2015.

### Outcome variables

Each elderly person in the survey was asked about his or her HMS utilisation in the past year. The main outcome variable, namely, HMS utilisation, was measured by five key indicators: (I) no HMS (1 if the elderly did not use any HMS, 0 otherwise); (II) health assessment (1 if the elderly used this service, 0 otherwise); (III) physical examination (1 if the elderly used this service, 0 otherwise); (IV) auxiliary examination (1 if the elderly used this service, 0 otherwise); and (V) health guidance (1 if the elderly used this service, 0 otherwise).

### Independent variables

The independent variables were divided into three categories: predisposing predictors, enabling predictors, and need predictors [18]. Predisposing predictors include age, gender, education, family size, and marital status. Enabling predictors include social medical insurance, private health insurance, household income, distance from the nearest primary health facility, service satisfaction score of village clinics, and service satisfaction score of township hospitals. Need predictors represent the potential needs of the elderly for HMS. We used “have chronic disease or not” to assess the needs of the elderly.

### Analytical methods

#### Concentration Index (CI)

CI is a popular method to measure the degree of socioeconomic-related inequality in a health variable, and is defined as twice the area between the concentration curve and the line of equality (the 45°line). In this study, we used CI to measure inequality of HMS utilisation for the elderly in rural areas of Henan, China. Standard CI was computed as follows [19]:

1 $$ {C}_I\Big(h\left|y\Big)\right.=\frac{2\operatorname{cov}\left({h}_i,{R}_i\right)}{\overline{h}}=\frac{1}{n}\sum \limits_{i=1}^n\left\{\frac{h_i}{\overline{h}}\left(2{R}_i-1\right)\right\} $$

where *h*_*i*_ is the outcome variable of *ith* individual, *R*_*i*_ is the fractional rank of *ith* individual in the distribution of household income, and $\overline{h}$ is the mean of *h*_*i*_. *C*_*I*_ is between − 1 and 1. If the *C*_*I*_ value is 0, no income-related inequality exists; if the *C*_*I*_ value is negative, the result variable is disproportionately concentrated in the relatively poor; if the *C*_*I*_ value is positive, the result variable is disproportionately concentrated in the relatively rich [20]. The standard CI requires that the outcome variable is on the same scale of income, that is, measured on a ratio scale [21]; if the outcome variable is binary, the standard CI needs to be revised. This study adopted the Erreygers’ modified index (*E*_*I*_) given by [19, 21, 22]:

2 $$ {E}_I\Big(h\left|y\Big)\right.=\frac{1}{n}\sum \limits_{i=1}^n\left\{\frac{4{h}_i}{\left({h}_{\mathrm{max}}-{h}_{\mathrm{min}}\right)}\left(2{R}_i-1\right)\right\}=4\left(\frac{\overline{h}}{h_{\mathrm{max}}-{h}_{\mathrm{min}}}\right){C}_I $$

where *h*_*max*_ and *h*_*min*_ are the upper and lower bounds of outcome variables, respectively.

#### Decomposition of CI

Although the measurement of CI provides an overall view of inequity in HMS utilisation for the elderly, it offers no explanation for factors that determine the inequality. Wagstaff et al. put forward that CI can be decomposed into the contributions of explanatory factors through the regression-based decomposition [20, 23]. Considering that the measures of HMS utilisation are binary variables, we used a modified decomposition method [24].

First, the regression model of outcome variables and related factors was established:

3 $$ {h}_i=\alpha +\sum \limits_{\mathrm{j}}{\beta}_j{x}_{ji}+{\varepsilon}_i $$

where *β*_*j*_ is coefficient of *x*_*j*_, and *ɛ*_*i*_ is an error term.

Second, the concentration index for *h* can be written as:

4 $$ {C}_I=\sum \limits_j\left(\frac{\partial \overline{E}\Big(h\left|x\Big)\right.}{\partial {x}_j}{\overline{x}}_j/\overline{h}\right){C}_j+{GC}_{\varepsilon }/\overline{h} $$

where $\frac{\partial \overline{E}\Big(h\left|x\Big)\right.}{\partial {x}_j}$ is the average marginal effect of *x*_*j*_,${\overline{x}}_j$ is the mean of *x*_*j*,_ *C*_*j*_ is the *CI* of the *j*-th explanatory variable(defined analogously to *C*_*I*_), *GC*_*ɛ*_ is the generalized concentration index for *ɛ*_*i*_, ${e}_j=\frac{\partial \overline{E}\Big(h\left|x\Big)\right.}{\partial {x}_j}{\overline{x}}_j/\overline{h}$ is the elasticity of *h* with respect to *x*_*j*_, indicating the weight of *C*_*j*_ in the total contribution of *C*_*I*_. All statistical analyses of data were performed on STATA 15.0.

## Results

The descriptive characteristics of all samples are listed in Table 1. Overall, the utilisation rates of the four services were low, with values of 18.67, 34.21, 33.29 and 21.38%, respectively, and more than half of the elderly (51.67%) had no HMS utilisation. The study population had an average age of 70 years, and consisted of 53.03% females. Over 76% of the elderly were married, and more than 64% had primary school and below education level. Based on the household income level, more than 20% individuals were the poorest. Nearly 40% of the elderly had chronic diseases. Almost all of the elderly were covered by social medical insurance, of which more than 90% were covered by the New Rural Cooperative Medical Scheme (NRCMS). However, only 3.92% of seniors were covered by the private health insurance. The average family size of each elderly person was 3.43, and the average distance between their residence and the nearest primary  health facility was 1.57 km. The elderly people’s satisfaction score of village clinic services (3.54) was higher than that of township hospitals (3.50).

**Table 1:** Characteristics of the study samples

Variable name	N/mean	%/S.D
Utilisation of HMS for the elderly		
No HMS utilisation	725	51.67
Health assessment utilisation	262	18.67
Physical examination utilisation	480	34.21
Auxiliary examination utilisation	205	33.29
Health guidance utilisation	300	21.38
Age	70.47	5.32
Gender		
Male	659	46.97
Female	744	53.03
Marital status		
Unmarried	21	1.50
Married	1075	76.62
Divorced	12	0.85
Widowed	295	21.03
Education level		
Primary school and below	909	64.79
Junior high school and above	494	35.21
Household income categories		
Poorest	286	20.38
Poorer	309	22.03
Middle	260	18.53
Richer	265	18.89
Richest	283	20.17
Social medical insurance		
New Rural Cooperative Medical Scheme	1276	90.95
Urban Resident Basic Medical Insurance	49	3.49
Urban Employee Basic Medical Insurance	34	2.43
Others	18	1.28
No insurance	26	1.85
Private health insurance		
No	1348	96.08
Yes	55	3.92
Family size	3.43	1.23
Distance from the nearest primary health facility	1.57	1.31
Any chronic disease		
No	843	60.09
Yes	560	39.91
Service satisfaction score of village clinic	3.54	0.86
Service satisfaction score of township hospital	3.50	0.83

The concentration indices of HMS utilisation are reported in Table 2. The result showed a statistically significant pro-poor distribution of the elderly with no HMS utilisation (*CI* = -0.0730, *p* = 0.0155). Significant pro-rich distribution was calculated for physical examination utilisation (*CI* = 0.0575, *p* = 0.0448) and auxiliary examination utilisation (*CI* = 0.0811, *p* = 0.0044). Although the negative *CI* values for health assessment and guidance utilisation were measured, their pro-poor effects were not statistically significant (CI = -0.0173, *p* = 0.4617; CI = -0.0213, *p* = 0.3900).

**Table 2:** Concentration indices of HMS utilization for the elderly

	CI(SE)	*p*
No HMS utilisation	-0.0730(0.0301)	0.0155
Health assessment utilisation	-0.0173(0.0235)	0.4617
Physical examination utilisation	0.0575(0.0286)	0.0448
Auxiliary examination utilisation	0.0811(0.0284)	0.0044
Health guidance utilisation	-0.0213(0.0248)	0.3900

The decomposition of concentration indices of the outcome variables is shown in Table 3. The main contributors to pro-poor distribution of no HMS utilisation were: household income, social medical insurance (NRCMS), family size, gender (female), age, marital status (married), and service satisfaction score of village clinics. Household income made the largest positive contribution to inequality (55.07%), which revealed that low-income elderly people were more likely to have no HMS. The family size was also a positive contributor to inequality (4.93%). Social medical insurance (NRCMS) made the largest negative contribution to inequality (− 21.23%), indicating that the coverage of NRCMS could reduce inequality. Moreover, age (− 2.19%), gender (female) (− 2.88%), marital status (married) (− 1.10%), and satisfaction with village clinical services (− 1.78%) were important contributors to reduced inequality. The remaining factors such as education, chronic disease, and service satisfaction with the township hospital made small contribution to inequality in no HMS utilisation (less than 1%).

**Table 3:** Decomposition of concentration indices of HMS utilization

	Concentration index	Elasticity			Contribution			Percentage Contribution(%)		
		No HMS utilisation	Physical examination utilisation	Auxiliary examination utilisation	No HMS utilisation	Physical examination utilisation	Auxiliary examination utilisation	No HMS utilisation	Physical examination utilisation	Auxiliary examination utilisation
Age	0.0018	0.8673^b^	−1.3265^b^	−1.2172^b^	0.0016	−0.0024	−0.0022	−2.19	−4.17	−2.72
Gender										
Male	Ref.									
Female	−0.0456	−0.0461	0.0749^a^	0.0758^a^	0.0021	−0.0034	− 0.0035	−2.88	−5.91	−4.32
Marital status										
Widowed	Ref.									
Unmarried	0.0045	−0.0022	− 0.0023	0.0001	0.0000	0.0000	0.0000	0.00	0.00	0.00
Married	0.0185	0.0418	−0.1494^a^	− 0.1525^a^	0.0008	− 0.0028	− 0.0028	−1.10	−4.87	−3.46
Divorced	−0.0095	0.0016	0.0005	0.0009	0.0000	0.0000	0.0000	0.00	0.00	0.00
Education										
Primary school and below	Ref.									
Junior high school and above	0.0067	0.0420^b^	0.0137	0.0209	0.0003	0.0001	0.0001	−0.41	0.17	0.12
Household income	0.2718	−0.1480^c^	0.1724^b^	0.2476^c^	−0.0402	0.0469	0.0673	55.07	81.57	83.09
Social medical insurance										
No insurance	Ref.									
New Rural Cooperative Medical Scheme	−0.0514	− 0.3012^a^	0.4216^b^	0.5220^c^	0.0155	−0.0217	−0.0268	−21.23	−37.74	−33.09
Urban Resident Basic Medical Insurance	0.0122	−0.0224^b^	0.0223^b^	0.0245^b^	−0.0003	0.0003	0.0003	0.41	0.52	0.37
Urban Employee Basic Medical Insurance	0.0218	−0.0017	0.0044	0.0071	0.0000	0.0001	0.0002	0.00	0.17	0.25
Others	0.0106	0.0024	0.0008	0.0001	0.0000	0.0000	0.0000	0.00	0.00	0.00
Private health insurance										
No	Ref.									
Yes	−0.0138	−0.0020	0.0050	0.0046	0.0000	−0.0001	−0.0001	0.00	−0.17	− 0.12
Family size	0.0494	−0.0732	0.1755	0.1621	−0.0036	0.0087	0.0080	4.93	15.13	9.88
Distance from the nearest primary health facility	−0.0036	0.0981^c^	−0.1395^c^	−0.1304^c^	− 0.0004	0.0005	0.0005	0.55	0.87	0.62
Any chronic disease										
No	Ref.									
Yes	−0.0070	−0.0530^b^	0.0762^b^	0.0666^b^	0.0004	−0.0005	−0.0005	− 0.55	−0.87	− 0.62
Service satisfaction score of village clinic	−0.0025	− 0.5187^c^	0.5956^c^	0.5267^c^	0.0013	−0.0015	−0.0013	−1.78	−2.61	−1.60
Service satisfaction score of township hospital	0.0011	−0.3386^b^	0.6382^c^	0.6767^c^	−0.0004	0.0007	0.0007	0.55	1.22	0.86
Residual					−0.0501	0.0326	0.0412	68.63	56.70	50.74
Total					−0.073	0.0575	0.0811	100	100	100

Notes:Estimation sample was 1403, ^a^, ^b^, and ^c^ indicate statistical significance at the 10,5, and 1%

The decomposition results of inequality in utilisation of physical and auxiliary examination showed that their main contributors to inequality were similar to those of no HMS utilisation. The largest positive contribution to the pro-rich distribution in utilisation of physical and auxiliary examination was household income (81.57, 83.09%), followed by family size (15.13, 9.88%). The largest negative contributor to inequality was social medical insurance (NRCMS) (− 37.74, − 33.09%), followed by gender (female) (− 5.91, − 4.32%).

## Discussion

The utilisation rate of HMS for the elderly was low in rural areas of Henan province and the inequality was pro-rich. In detail, no HMS utilisation had high proportion among the poor, and utilisation of the physical and auxiliary examination had high proportion among the rich. However, the absolute values of *CI* for no HMS utilisation, physical examination utilisation, and auxiliary examination utilisation were less than 0.1, which implied that the inequality in HMS utilisation was not a serious problem. The results also found that the utilisation of health assessment and guidance was pro-poor, but the results were not statistically significant. Furthermore, we proved the contribution of different factors to the inequality results. The main factors included household income, family size, social medical insurance, and so on.

The pro-rich finding of HMS utilisation for the elderly in rural areas of Henan is consistent with some studies but different from others [15–17]. The utilisation of HMS varies in different regions or countries possibly due to differences in the health management system and financial investment [25]. The utilisation is also affected by the health status of the elderly, demographic characteristics, etc.

The results of the CI decomposition revealed important information. Household income was considered to be the biggest contributor to unequal utilisation of HMS, and income gap was not conducive for low-income elderly people to obtain HMS. This result is consistent with previous research [26, 27]. Although HMS for the elderly is free, it does not seem attractive to the poor. Regardless of the type of services they use, once they get sick, economic factors play an important role in deciding whether to receive treatment [28].

NRCMS made the important contribution to the reduced inequality in HMS utilisation. To achieve the goal of health care for all, the nationwide social medical insurance system was implemented in China, and NRCMS was practiced in its rural areas. Evidence shows that the expansion of NRCMS coverage can reduce the medical expenses of old people and improve their health service utilisation, especially for low-income elderly people [29]. Therefore, the coverage of NRCMS reduced inequality in the utilisation of HMS.

Additionally, we found that social–demographic characteristics such as family size, age, gender, and marital status had important effects on inequality in HMS utilisation, but the direction of action was inconsistent. First, family size increased the uneven distribution of HMS. Other members of the family can provide active support for the elderly to use HMS, and extended families are more likely to be concentrated among rich families. Therefore, family size increased inequality in HMS utilisation. Second, age, gender (female), and marital status (married) contributed to the reduced inequality in HMS utilisation. Women are more likely to receive health services due to their special psychological and physiological characteristics [30]. Consequently, gender (female) reduced the income-related inequality in HMS utilisation. However, the reducing effect of age and married status on inequality in HMS utilisation could be attributed to the negative elasticity between them and HMS utilisation. In fact, the oldest and married seniors are more concentrated among high-income families. This group has a higher demand for HMS, and the existing management services do not match their needs. Therefore, their HMS demand has declined, which reduces the pro-rich inequality in HMS utilisation.

Finally, for the elderly, the improved service satisfaction with village clinics could reduce inequality in HMS utilisation, while the improved service satisfaction with township hospitals could increase inequality in HMS utilisation. This finding deserves the attention of policy makers. At the bottom of the three-level health service network in rural areas, village clinics are the most convenient institutions for rural residents to seek health services [31]. Furthermore, village clinics mainly provide services for a small-scale or fixed population, which is more suitable for communication between the elderly and doctors. Therefore, the more satisfied with rural doctors the elderly are, the better it will be to expand the coverage of management services. Usually, only one hospital is set up in a township to provide services for all residents, causing great inconvenience to the elderly who live far away and have inconvenient transportation. In fact, most of the elderly living in remote areas belong to low-income group. Therefore, we speculate that when service satisfaction of the township hospital increases, the HMS utilisation for the rich elderly people will be more stimulated.

This study has several strengths and limitations. First, we expanded current studies on inequality in HMS utilisation. Second, the design of multiple outcome variables provides a better understanding of the similarities and differences in inequality in utilising each service. Third, the decomposition of CI provides a reference for policy makers to reform HMS for the elderly. However, a significant limitation of the paper is that the findings based on the samples selected from rural Henan province may not be fully applicable to other areas given the differences in economic levels and social characteristics. Another limitation is the recall bias because some seniors or their families could not remember much about the use of HMS. Additionally, only 1 year of HMS utilization for the elderly was observed. Therefore, we could not further investigate the change in the uneven distribution of HMS during the health care reform in rural Henan.

## Conclusions

Our study holds the idea that the degree of inequality in utilisation of HMS for the elderly is low, but inequality disproportionately favors the rich in rural Henan, China. The inequality decomposition analysis suggested that NRCMS is the biggest contributor to the reduction of inequality, while household income is the biggest contributor to the increase of inequality. Meanwhile, social-demographic characteristics such as family size, age, gender and marital status are important in explaining inequality in HMS utilisation for the elderly. The elderly’s satisfaction with village clinics and township hospitals plays the opposite roles in the inequality of HMS utilization. Although HMS for the elderly is provided free of charge, its accessibility remains unequal due to various factors. Under the trend of population aging, this inequality should deserve our adequate attention. Therefore, policy makers should adopt effective interventions to resolve the contradiction between various factors and HMS utilisation for the elderly, and redress inequality in HMS utilisation for the elderly, rather than simply providing an equal process.

## Data Availability

The data that support the findings of this study are available from China urban and rural health survey and research center of Xinxiang Medical university, but restrictions apply to the availability of these data, which were used license for the current study, and so are not publicly available. Data are however available from the authors upon reasonable request and with permission of China urban and rural health survey and research center of Xinxiang Medical university.

## Abbreviations

HMS: Health management services
HRRHS: Henan Rural Residents Health Survey
CI: Concentration index
NRCMS: New Rural Cooperative Medical Scheme
